# The search for successful welfare technology (WFT) implementation: Norwegian municipalities implementing a WFT coordinating role-a qualitative study

**DOI:** 10.1186/s12913-025-13047-3

**Published:** 2025-07-01

**Authors:** Gunn Hilde Rotvold, Marianne Vibeke Trondsen, Anne Grete Sandaunet

**Affiliations:** 1 School of business and economics, UIT- The Arctic University of Northern Norway, Tromsø, Norway; 2 Norwegian Centre for E-health Research, University Hospital of North Norway, Tromsø, Norway; 3 Department of Social Sciences, UIT - The Arctic University of Northern Norway, Tromsø, Norway

**Keywords:** Welfare technology implementation, Municipal administrations, Coordinationg roles, Change management theory

## Abstract

**Background:**

The implementation of welfaretechnologies (WFT) is currently taking place across European healthcare delivery. The implementation is accompanied by research demonstrating further that digital health implementation in healthcare is a complex and challenging process, and more attention to the management of large-scale implementation of WFT is needed. This article addresses the question of how to implement WFT and explores the implementation of welfare technology coordinating roles currently taking place in several Norwegian municipalities.

**Methods:**

The article is based on a qualitative study in five Norwegian municipalities. Thirty-two semi-structured individual interviews were conducted in 2021 with a strategic selection of administrative participants involved in the WFT implementation process. A thematic analysis was done following Gioa’s model for structuring the empirical material. The analysis evolved as an iterative process, pending between empirical codes and concepts, themes, and aggregated dimensions.

**Results:**

The main finding was that the implementation of a WFT coordinating role was experienced as challenging by the municipal administrations. We identified three categories that provided further contents to this main finding: (1) the challenge of delimiting the role (2) the challenge of gaining commitment from other actors in the organisation (3) the challenge of locating the role in the organisation.

**Conclusions:**

Based on the findings, this study demonstrates further how WFT implementation can be a challenging conduct. The study also contributes to more insight into how such challenges can be constituted and suggests that further attention should be paid to (i) how WFT change designs relate to conflicting interests and heterogeneity in the organisation and to (ii) how WFT change interventions interact with underpinning processes and structures in municipal organisations.

**Supplementary Information:**

The online version contains supplementary material available at 10.1186/s12913-025-13047-3.

## Background

Welfare technology (WFT), also referred to as assistive technology [1] or telecare [2], is a broad and loosely defined concept that encompasses a range of digital technologies introduced into healthcare delivery. The implementation of WFT is currently taking place across European healthcare delivery [3], and according to the World Health Organisation [4], assistive technology represents a key component in European health and care services, intending to improve individual well-being, independence, inclusion and participation. The implementation of WFT also represents a key component to sustainability of a health and care system which is threatened by a well-known demographic change and lack of human resources [5]. A body of research accompanying the ongoing implementation of WFT shows that the road to implementation can be bumpy, for example by illustrating that WFT must fit into the healthcare organisation’s culture, infrastructure, working practices, expertise and management practices in order to realise its potential [6–9]. As such, an overall picture of digital health implementation as a complex and challenging process [2, 10–18] is confirmed by research on WFT implementation.

In response to this emerging picture, and also corresponding to the broader research area of digital health implementation [19–21], the management of large-scale implementation of WFT is among the areas suggested to be in need for further attention [22–25]. Baudin et al. [22] direct attention to a modest base of research literature on policy features and guidance surrounding WFT implementation. In his contribution, Aaen [25] directs attention to WFT innovation and to an *“emerging and fragmented”* (25: page 11) research literature on this area, “*charachterised by many unsolved debates*,* speculations*,* and competing concerns*” (25: page 11). In a systematic analysis of the research literature [25] he identifies eight competing concerns needing to be considered in relation to large scale implementation of WFT. Among these are considerations on decision making structures, governmental participation and how to approach implementation of WFT. Empirical studies that focus on exploring and expanding on the competing concerns are also called for. Against this background, this article aims to provide further insights into the management of WFT implementation by directing particular attention to concerns on how processes of implementing WFTs are organized. The article is based on an exploration of the implementation of WFT coordinating roles which is currently taking place in several Norwegian municipalities.

Coordination can be defined as “*the purposeful alignment of tasks and efforts of units or actors in order to achieve a defined goal*” (26: page 4) and is argued to represent a pivotal instrument in contemporary public administration [26].The creation of a WFT coordinator role in Norwegian municipalities also echoes a development seen in other municipal areas, such as corresponding initiatives in the implementation of Information and Communication Technology (ICT) in primary education [27, 28], sustainability strategies [29] and strategies on public health [30]. We find that research on such initiatives is scattered, but some studies [27, 30] point to an ambiguity accompanying the conduct of coordinating roles, referring to situations where the responsibility, authority and expectations are unclear and poorly defined. Relating to this broader picture, further research attention to the management of municipal coordination roles is brought into focus. In this article, the attention is particularly directed at the implementation of municipal WFT coordination roles, and we investigate the following research question; “*How is the process of implementing a welfare technology coordinator role unfolding in Norwegian municipalities?”* The article is based on a qualitative study with interviews of WFT coordinators and managers at different levels in five Norwegian municipalities.

### Theoretical framework

Our analysis draws on insights from the research literature on change implementation. From this point of departure, the implementation of a WFT coordinating role implies that a new function intervenes with existing administrative processes that are supporting and enabling municipal health care provision, carrying a particular responsibility for aligning task and efforts contributing to facilitate the implementation of WFT. This step in the process of implementing WFT thereby represents a situation of planned change, which is a core subject of attention in the change implementation literature [31].

A prominent approach in analyses of change implementation, and an important starting point for this analysis, is to direct attention to the change design that is enacted during the implementing process [31–33]. In this literature, a divide between top-down and to bottom- up oriented designs is common. A well established approach [32] is also to describe the contents of top-down oriented designs as “theory E” and the contents of bottom up oriented designs as “theory O”. This conceptualisation is here considered as helpful for the effort to grasp how the process of implementing a WFT coordinator role unfolded. The analytical framework brings further details to the purpose and means of each of the two approaches by suggesting how leadership, focus, planning, motivation and use of consultants are provided with contents [31, 32]. When commiting to “theory E” in change implementation for example, the change is driven from the top and through the help from consultants, or change agents, entering a role as experts. When committing to “theory O”, the change is emerging and participative, characterised by experiementation and including consultants who tend to rely on social influence (i.e. “champions”) instead of the influence provided by formal positions [34], emerging spontaneously and informally within an organisation.

Importantly, and both traced in the research literature and in debates on the practical and political level, bottom-up oriented change designs (illustrated by “theory O”) have gained more attention. This development can be argued to build on the complexity recurrently demonstrated in studies of change implementation, and on debates on the contents of change that have brought attention to its micro-oriented and heterogeneous character [35, 36].

The main point of Beer and Nohria [32] however, and further developed by Leppitt [33], is that an integrated change design should guide implementation processes – implying a combination of “theory E” and “O” in the terminology used here. This view is interesting in relation to more recent organisational perspectives proposing that paradoxes and conflicting needs are salient and persistent in organisations [37]. Similar arguments are also included by Aaen [25] in his suggetions of theoretical frames to be used in further studies of large scale WFT implementation. Based on such perspectives, more integration of the directive appraoch inherent in “theory E” change designs can emerge as a balanced response.

A last point needed to be included in considerations of change implementation is a closer reflection on how to account for complexity, as different conceptualisations of the depth of change are brought to the fore (see Heraclaous [35] for a further outline). It means that the literature directs attention to different levels in the organisation in need of attention during change implementation processes. A common approach [35] is to focus on resistance and to emphasise the motivation and values among those exposed to a change intervention. Others [35, 38, 39] extend the attention to include ongoing work processes and structures of the receiving organisation, and indicate that change designs need to address this deeper level of the organisation for change to succeed.

## Methods

The article is part of a larger qualitative study aiming to explore how the implementation process of WFT unfolds from different perspectives.

### Study setting

In Norwegian municipalities, the processes of implementing WFTs is moving from a “project mode” to integrating WFT into regular health care delivery. In 2022, eight years after the introduction of a national programme for WFT activities approximately 80% of the municipalities had initiated WFT projects supported by the program. The national program [40, 41] was introduced to support and guide the municipalities’ implementation processes. Through this program the municipalities were provided with networking facilities and could apply for funding to establish and carry out WFT pilot project in order to gain WFT experience and knowledge. Based on the experiences of the participating municipalities, the program developed national recommendations on WFT implementation, while the choice to decide which steps to take during the implementation process was assigned to each municipality. This division of responsibility complies with decentralisation, which is argued to characterise interactions between the national and county/municipal levels of welfare and health care in Norway and Scandinavia [30].

The assignment of a local “project owner” chosen by the municipalities represents one proposal in the national recommendations [42], and creation of WFT coordination roles has emerged as a common take on the further process of WFT implementation on municipal level.

### Recruitment and sample

The selection of municipalities for this study was based on information from the Norwegian Association of Local and Regional Authorities and an overview of the implementation of WFT in Norwegian municipalities that was available. The main inclusion criterion for the study was municipalities that had started implementing WFT into their regular health and care services. In addition, to cover a broad spectrum of experiences, we selected municipalities that represented variations in size and geographical regions. A written invitation was sent to the health and care managers in five different municipalities, and all confirmed their participation and appointed a contact person for further collaboration. All the five municipalities worked with technologies such as Digital safety alarms (DSA), Electronic medication dispensers (EMD), GPS tracking, and Surveillance technology (ST). In all municipalities, WFT implementation processes are taking place at various institutions and in the home service. One of the municipalities had postponed the implementation in the home service awaiting the upgrade of the network infrastructure.

The contact person from each of the participating municipalities collaborated regarding recruiting relevant participants for individual interviews. The aim was to recruit participants involved in the implementation of WFT on an administrative level in the municipal organisation. Based on information from the municipalities, the first author sent a written invitation to 54 selected persons and invited them to participate in the study. The total sample consists of 44 people including managers at different levels, coordinators, e-health counsellors, local politicians and end-user representatives in all five municipalities. This article is based on 32 interviews conducted with 23 managers at different levels and the nine coordinators in the sample. Actors in these positions are expected to be crucial contributors to the implementation of the role, making first hand experiences to how the implementation process is unfolding. Two coordinators in one of the municipalities were replaced during the study period, and the new persons were included in the sample. The coordinating role is usually located next to the line in the organisation, either in the staff department or in one of the municipality’s health service units. One of the municipalities created a WFT coordinator role at both staff and operative level. The five municipalities had different organisational structures with two or three administrative levels. We define middle managers to include individuals with *strategic* managerial positions, while first-level managers include those with *operating* managerial positions. The interviews with 26 women and six men in the sample were considered as relevant for this article. A large predominance of women means that for reasons of anonymity, gender is not shown in the table and for simplicity we refer to everyone as women. An overview of the municipalities, technologies in use and the study participants are presented in more detail in Table 1.

**Table 1:** Sample of municipalities and participants

Sample of municipalities			Sample of participants			
	Population	Technology	WFT coordinators		Middle managers	First-level managers
			Share	Location		
M 1	> 25 000	DSA, GPS, ST, EMD	2 (50%)	Health service unit	1	3
M 2	5 000	DSA, ST	1 (50%)	Health service unit	1	5
M 3	5 000	ST	1 (100%)	Health service unit	1	2
M 4	> 25 000	DSA, GPS, ST, EMD	1 (100%)	Staff	1	6
M 5	> 100 000	DSA, GPS, ST, EMD	4 (100%)	Staff/Health service unit	1	2

### Interviews

The first author conducted the individual interviews in April-November 2021, using a semi-structured interview guide to structure the dialogues. All participants were asked about their role and the processes they had been through, their expectations of WFT and their experiences associated with implementing WFT. Due to the COVID-19 restrictions, only 10 out of 32 interviews were conducted face-to-face in suitable premises in the respective municipalities. The remaining 22 were carried out on video using Microsoft Teams. All the interviews lasted between 50 and 60 min. The interviews were digitally audio-recorded and fully transcribed afterwards.

### Analysis

The analysis consists of a thematic content analysis based on Gioia et al.’s [43] approach to grounded theory formulation and model of structuring data. In this model, the structuring of data is built up from first order (informant based) to second order (research based) concepts [43]. Against this backdrop, our analysis evolved into an iterative process, in which the empirical codes and our concepts, themes and dimensions were clarified. As an initial step, the first author read and coded the transcripts inductively using the NVivo analysis software program. In the next step, the codes were reviewed, discussed and interpreted in collaboration with the other authors. Together, all authors identified several prominent themes in the material. Through this process, the creation of the WFT coordination role emerged as a key topic for this article. In further analysis, we investigated the descriptions identified as relevant for analysing the implementation of the WFT coordinating role. We clustered basic codes separately from managers and coordinators, grouped them and considered relevant theories related to change implementation. This process resulted in three categories of challenges in how managers and coordinators described the unfolding of a WFT coordinator role in Norwegian municipalities: delimiting the role, gaining commitment from other actors in the organisation, and location of the role.

The structure of the analysis of the empirical material in this article is presented below in Table 2.

**Table 2:** Structure of the analysis

“How is the process of implementing a welfare technology coordinator role unfolding in Norwegian municipalities?”			
Examples	1 st order concepts	2nd order themes	Aggregated dimensions
• No available guidelines for the function • WFT- coordinator tasks defined by national guidelines for implementation of WFT • The coordinators were drawn in all directions • A point of contact • WFT competence dissemination	• An extensive character of the role • An unclear role	The role was challenging to delimit	• Inaccurate arrangement of change implementation • Insufficient accountance for existing processes and structures
• The coordinators are in the forefront of WFT knowledge • The experience of complex technology • Acting like sellers • Poor anchoring both upwards and sideways • Managers express uncertainty • Delegated responsibility • The experience of Loneliness • Trust and support in the national WFT program	• Facing declined motivation among health care professionals • Little support from managers	It was challenging to gain commitment from other actors in the organisation	
• Ongoing discussions about location • Closeness to service providers • A point of contact for the entire organisation • No right place for the role • Coordinators’ perceptions of tasks • Managers’ perceptions of Tasks	• A prevailing uncertainty about location • Limited outreach across departments and units	The role was challenging to locate	

## Findings

As an overall topic, the establishment of the coordinator role was something that many participants in this study referred to as a natural first step for successfully implementing WFT in municipal health and care services. However, the main impression is that WFT coordination is a challenging arrangement and that this character of the process dominated how the implementation of the WFT coordinator role unfolded across municipalities and across the different positions. We identified three main categories of challenges: (1) the challenge of delimiting the role (2) the challenge of gaining commitment from other parts of the municipal organisation (3) the challenge of locating the role in the organisation. We will outline these categories in the following subsections.

### The challenge of delimiting the role

The first challenge relates to the content of the WFT coordination role and the challenge of delimiting it. The first aspect of this challenge that we will emphasise rises from the impression that the role has become extensive. One of the experienced coordinators, described the content of the role as a position that has “*quite a wide board with various tasks.*” The same coordinator commented the role as essential to the implementation process, bringing to the fore the introduction of new technologies and new ways of working. However, she also underscored the extensive character of the role and described it as a central point of contact between many different actors and as responsible for the training and dissemination of knowledge:

*“Then*,* it is a point of contact for all services in the municipality. One is often the point of contact for other stakeholders or partners. These may be suppliers*,* and they may also be with management. Moreover*,* one is also the administrator in the municipality who ensures that all employees have access to the tools they need to perform their work tasks within welfare technology products. Then one is also responsible for training and ensuring everyone receives the expertise they need.”*

Several of the managers also contributed to the impression of an extensive role. They expressed concerns about the considerable need for WFT competence in the organisation and presented the dissemination of WFT knowledge as an essential part of the coordinating role. Additionally, some middle managers considered the coordinator role important for ensuring a point of contact for WFT in the organisation.

As a second aspect, we will emphasise that in addition to the impression of an extensive role, coordinators and managers referred to the WFT coordinator role as unclear. Some managers said that the mandate of the role needed to be more clearly described, and several coordinators stated that they needed more precise directions on the content of the role. As described in the study setting, creating the coordinator role was not imposed by the national program but a solution chosen by the municipalities. The role was a municipal matter, whereas the content of the role had to be developed by each of the municipalities. One of the coordinators expressed that “…*it is not really well defined what my tasks are.”* She also commented that she and the other coordinator in her municipality worked to define the role and cover the necessary steps in the implementation process. She also explained that another reason for defining the role was to prevent the coordinators from being “*pulled towards very different directions.*” In this situation, some of the coordinators found it difficult to prioritise which tasks they should work on.

It also belongs to the story that a written role clarification for the WFT coordinator role existed only in one of the municipalities due to a job announcement and the appointment of a new coordinator. In this situation, one manager assigned the previous coordinator to describe the role due to her experience with the WFT coordinator role.

In summary, the impression of a role that was difficult to delimit draws on descriptions of undefined and extensive tasks and a lack of more precise directions for the content of the role.

### The challenge of gaining commitment from other actors in the organization

The second challenge identified was gaining commitment from health personnel, managers from other health service units, and others in the organisation. The first aspect of this challenge concerns the commitment from health service personnel. Some coordinators said they faced difficulties motivating health service personnel to adopt and use WFT. One of them explained:

*“The technology is presented as much simpler than it is. It requires much adaptation. Employees lose motivation*,* creating problems for those of us who have to introduce this.”*

Several coordinators said they tried convincing health personnel about the positive effects of WFT. One of them described her performance as a “seller” because she felt she had to persuade health personnel to introduce WFT in the health and care service:

*“I feel very much like a seller…because one often comes with a technology that one has to convince the recipient that this is good for you*,* you have to use it. And then one meets a bunch of nurses who do not want to be convinced.”*

The second aspects we notice as indicative for difficulties in gaining commitment to WFT implementation is related to expressions about lack of support from decision making managers. Such utterances were uttered by both coordinators and first line managers working close to the WFT coordinators. Some of the coordinators described the function as lonely. One of them expressed:

*“I feel a bit alone with a specialised knowledge that few others understand.”*

Another coordinator with considerable experience gave a similar description and explained that being alone with WFT competence, without supporting surroundings, could give a feeling of loneliness. A third coordinator emphasised that the managers’ commitment to the WFT implementation should be more visible to the organisation, which could support the coordination work. Lastly, some coordinators also expressed that they wanted the managers to be more aware of the coordinator’s need for support and resources to ensure the implementation process.

Some first-level managers working close to the coordinators indicated support to the coordinators by calling for demands to put more pressure on the WFT implementation. These first line managers described experiences of weak alignment both sideways and upwards in the organisation. They also expressed that such a lack of commitment made them unsure whether the rest of the organisation would provide more effort and pressure on the implementation process. One commented, “*You have not understood what you have started.“*, and another expressed that:

*“Welfare technology is difficult for me because I do not quite have management support.”*

A further point we notice as indicative for difficulties in gaining commitment to WFT implementation is related to the impression of weak alignment. This impression was reinforced by both first-level managers from other departments and middle managers who said that they had lost their commitment to WFT during the implementation process. For example, more knowledge about WFT was called for, and some also commented that they found it difficult to engage because they experienced WFT implementation as complicated. They felt that they lacked the necessary expertise to fully commit to WFT implementation.

A middle manager expressed that she was surprised by the complexity of the WFT implementation and said:

*“What has surprised me the most is*,* how difficult this has been. When we started*,* I did not think this would be*,* or become*,* so difficult.”*

Some middle-managers also said they had delegated the responsibility for the WFT implementation process to the WFT coordinator. They described this delegation of responsibility as an effort to ensure progress.

In sum, the participants directed attention to a lack of commitment to WFT implementation in the organisation. The coordinators said they faced difficulties in motivating health service personnel to maintain commitment to implementing WFT. Managers who didn’t work closely with the coordinators expressed that they needed more WFT expertise to fully engage, and delegated responsibility to the coordinators.

#### The challenge of location

The third challenge we identified is related to the WFT coordinator’s organisational location. We noticed that the WFT coordinators participating in the study were located differently in the municipality’s organisational structure. The first aspect that underpin location challenges is therefore linked to our interpretation of a prevailing uncertainty about where the coordinators should be located. In one of the municipalities, the WFT coordinator role was handled by two people, each in a 50% position. These coordinators carried out the coordinator role from their respective locations. In one of the other municipalities, the WFT coordinator was first placed in a health section and then moved to the staff department. Several of the participants, independent of location, expressed uncertainty about where to place the WFT coordinator. One coordinator voiced the situation:

*“We did not know how to organise welfare technology. Moreover*,* we were placed in many units with managers with different understandings of welfare technology.”*

Several WFT coordinators mentioned that the role’s location affected their working tasks, which brings us to the second aspect of the location challenge; the availability across departments and units. One coordinator who were placed in one of the home care units, said she worked closely with the service providers in her belonging unit, acting like a super user, but faced challenges in being available to the entire organisation. She explained that the unit funded her position and that she felt expectations from the manager to prioritise internal tasks in the unit.

Moreover, some of the other coordinators also commented that the reach of the role across units depended on the manager’s understanding of WFT and whether the manager was orientated beyond their own department. One of the first-level managers who hosted the coordinator role in a home care unit underlined the value of having the WFT expertise close to the professional practice to be able to integrate WFT into the service:

*“However*,* we need the expertise locally in the department if we are to achieve something*,* and then there may be people we already have who we might want to develop further and become good at welfare technology.”*

Many first-level managers who hosted the coordinator position, expressed the value of having the coordinator located in their unit because the coordinator contributed to understanding the units’ needs, enhancing employee motivation and cooperation in the WFT integration. However, one of the first-level managers commented on how she revised her viewpoint on the location of the WFT coordinator after experiencing less availability for the position when she experienced a shift of unit. She underlined that, without the coordinator in her unit, she found it challenging to obtain access to WFT expertise and argued for the location of the position at a higher level in the organisation:

*“But now that I have changed focus and see things from a different role or perspective*,* we would have been better served by having the coordinator in more of a staff role. So*,* it is not the case that when someone gets swallowed up in some operation you are tied to where you belong.”*

Most middle managers expressed both advantages and disadvantages regarding the coordinators’ locations. In this respect, they highlighted the dissemination of knowledge as an important task, which, in their perspective, made it relevant for the coordinators to be located out in the units, paying attention to tasks relevant to the service providers. On the other hand, the middle managers also expressed that they wanted to have a point of contact higher up in the organisation across the department structures.

In summary, the participants described the localisation of the coordinator as an ongoing discussion on how to find an appropriate location for the position so that it was available to the entire organisation. The location of the coordinator role appeared to be a dilemma and effected the reach of the role.

## Discussion

This study aimed to gain further insight into how to organise WFT implementation by exploring how the process of implementing a WFT coordinator role in five Norwegian municipalities unfolded. The shared understanding about the creation of this role as a natural step forward is indicating that a key criterion for succeeding with change implementation [44, 45] is present. Overall, however, the complex and challenging conduct of implementing a municipal WFT coordinator role is further demonstrated by this material, supporting previous contributions on the implementation of digital health technologies [2, 10–21]. In the following, we discuss the three categories of challenging WFT implementation identified in this material and use change implementation literature to reflect on how they were constituted.

### The WFT coordinator role remains blurry

The first category highlights the WFT coordinating role as challenging to delimit. This interpretation relies on the impression that the role is experienced as extensive and unclear. As commented in the findings, the WFT coordinators conveyed a feeling that it was difficult to prioritise between tasks and that they should be everywhere all the time, while the unclear character is brought to the fore by uncertainty among the coordinators about where to focus. It is also interesting that some coordinators stated that they had to rely on the national recommendations when addressing tasks that the municipalities should focus on in their implementation processes, as further local structuring of the content had not taken place. The unclear character of the coordination role which is emerging from this material then seems to resonate with the ambiguity demonstrated in previous research on the creation of coordination roles at other municipal areas [27–29]. Further, and based on their study of coordination practices in 12 European countries, Lægreid et al. [26] direct attention to a similar situation, demonstrating that in many cases, coordination is introduced without a general master plan and conducted in a “trial and error” fashion that does not necessarily have positive results.

Relating this finding further to the change designs presented in the theoretical section we argue that “theory O” [32] is reflected, and particularly the non-programmatic and emerging character of the planning dimension integrated in this design. However, in their proposal of conducting a combination between “theory E” and “theory O” as the road to facilitating change, Beer and Nohria [32] include “theory E” and suggest that managers also should “*focus on planning the change process”* (32: page 27). Accordingly, a more active management of the implementation process than integrated in “theory O” can be called for, for example by the integration of a “well-sequenced” change effort being included in “theory E” [32]. It was characteristic for the municipal administrations studied here that preparing activities to provide content to the coordinating role, such as organised meetings and discussions, was not included in the process. In the case in which a job description was developed, for example, this effort to fill the function with content emerged as response to a situation in which one coordinator left the job and a new person was needed, not as part of planned activities arranged to contribute to providing content to the coordination role. As such, questions can be raised whether an outsourcing [21] of the implementation process is taking place.

On this backdrop, our argument is that an *inaccurate arrangement* of the change process contributed to the challenge of delimiting the coordinator role.

Our material does not provide indications about which managerial levels in these municipalities being in need to be addressed when it comes to planning activities. However, the material reminds us that an approach to the WFT implementation process as non-programmatic and emergent by involved managerial levels can lead to unclarity and considerable effort for those trying to identify the relevant tasks of the coordinator’s role.

A point made by Greenhalgh et al. [21] is thereby brought into focus. These authors direct attention to “*a mismatch between work-as-imagined and work-as-done*” (21: page 12) and argue that decision-makers and change planners tend to assume that issues are more predictable and controllable than those working close to front-line staff. Based on this material, we consider the difficulty related to prioritise between tasks is illustrative for such complexity at operative level, and as a reason to argue that elements of “theory E” could be part of the change design.

### Challenges in building organisational commitment

Our second category concerns challenges in gaining commitment to WFT when the WFT coordinators located in the units were interacting outside the unit to which they belonged. These challenges were brought to the fore by WFT coordinators expressing difficulties related both to motivating healthcare workers and receiving support from both their own managers and managers in other units. To our knowledge, similar challenges of gaining commitment are not described in the research on coordination roles in other municipal areas. Our suggestion is that this finding indicates that it can be difficult to enact a role as champion in these relations, and which is one of the elements emphasised in a “theory O” approach [32]. Based on our discussion of the challenge of delimiting the coordinator role, a “theory O” approach appears to be initiated when these municipalities embark on the implementation of the WFT coordinating role.

This study is not including interviews with health care workers, and we don’t have indications on how feelings among the coordinators of being a salesperson will find resonance among the health care workers from other units interacting with the coordinators. However, the coordinator descriptions bring attention to the notion of resistance to digitalisation projects [46] and to welfare technology [47] within health care services. In their study, Nilsen et al. [47] identified technology resistance as one of four primary forms of resistance occurring in relation to technology implementation, describing this resistance as emerging from complicated technology characteristics and various perceived threats in municipal health care. According to these authors, technology challenges healthcare workers’ perception of predictability, professionalism and expertise - and affects their motivation to utilise technology. As such, technology resistance could be considered as constitutive for the challenge of gaining commitment form health care workers identified in this material.

On the other hand, literature on change management [9, 35, 48] also provides reasons for interpreting the lack of commitment to WFT implementation among health professionals as illustrations of difficulties in integrating technology in the established working practices. From this perspective, the role of existing work practices and routines is further emphasised and broadens the attention beyond the resistance for using WFT among the involved actors. The perspective is illustrated and deployed in studies of WFT implementation in professional work [6–9]. Related to our context it is possible to argue that such arguments direct attention to *insufficient accountancy* for the depth of complexity into which the WFT coordinating role is inserted.

In the wake of this latter perspective, it is our argument that the coordinators’ attention to the difficulty of gaining commitment from managers also identified in this material needs particular attention. The descriptions of lack of managerial support for example, could imply that a role as champion [32] was not clearly enacted by the managers. Some descriptions from both first-level and middle managers presented in the result section indicate that a lack of participation was acknowledged by managers, for example indicated by the managers stating they found it difficult to engage in the process. It is possible to interpret such findings as a further signal of outsourcing [21] of WFT implementation by managers. However, following the perspective presented above, accountancy for existing work practices and routines at managerial levels should also be included in efforts to understand how outsourcing of WFT implementation is constituted. Further insights into how the implementation of WFT technologies affects the tasks and work practices of managers are therefore necessary, not at least due to managers being important facilitators of change [49]. In the wake of stating that it was difficult to engage in the process, and as further demonstrated in the result section, several managers also described WFT as more complicated and time-consuming than expected. They expressed surprise about how difficult the implementation work turned out to be and they felt they lacked the necessary expertise to fully commit to WFT.

### Silos and the coordinator’s reach

Our third category concerns challenges related to the localisation of the coordinating role. The concerns among WFT coordinators on the limited availability of the function outside the unit they belonged to illustrate these experiences. WFT coordinators belonging to units at the operative level of the municipal organisation appeared to be of high value to these units. At the same time, there were also utterances among the coordinators directed at a limited outreach of their support across units and departments. This situation adds to the challenge of gaining commitment in the sense that the WFT coordinators also convey an experience of a limited “outreach” of the coordination role. Consequently, the coordinators tended to prioritise tasks their immediate manager and colleagues requested, which might lead to the WFT activities being concentrated in separate parts of the organisation. This finding resonates with Quitzau et al. [29] who found that sustainability coordination was enacted differently depending on location of the coordinator. They describe how a decentralised location of the position establishes a robust commitment to *“narrow action points”* and how a centralised coordinator delegates more “*vague action points”* in which coordinators are enabled to participate broadly throughout the entire organisation (29: page 1). The limited availability of the coordinator function expressed by WFT coordinators in decentralised positions in this material resonates with a suggestion of narrow action points, or the emergence of a “myopic” WFT coordinator role. We consider this as an interesting finding, while also being aware that similar expressions were not provided by coordinators located at a centralised level in the municipalities. The argument that can be raised on basis of the utterances by coordinators at a decentralised level, is that this finding provides associations to the broader phenomenon of siloisation [26] as constitutive for the difficulties related to locating the WFT coordinator role.

Siloisation refers to a phenomenon where different departments or teams operate in isolation and is argued to be present in the public sector [26]. According to Lægreid and Rykkja [50], siloisation implies that traditional public administration is alive and kicking and backfiring on coordination. Based on this study, we are not in the position to have sufficient insight into the presence of siloisation at the municipal level in Norway. Heartley [51], for example, suggests that a traditional (hierarchical) public administration represents one of three governance paradigms currently being present in the public sector in Europe and that both distinct forms and combinations of these regimes can be present in different contexts of public administration. Therefore, it is important to be careful with assumptions of siloisation as a barrier in municipal WFT implementation, at the same time as it is important to raise awareness of the structures or different paradigms that can be present and how they interact with change efforts in public organisations. Again, *insufficient accountancies* for existing processes and structures can be argued to underpin the challenges encountering the implementation of a WFT coordinating role.

In sum, the choice of change designs and particularly a close consideration of the interaction between the WFT coordinator function and underpinning processes and structures in the municipal organisations are brought into focus by this study. We argue that by relying on a bottom-up oriented change design in this process, exemplified in this study by “theory O” [32], and which seems to be the chosen design in the municipalities studied here – the management of conflicting interests and heterogeneity [37] being activated by the change, risk being a challenge that is left to the operative level in the municipal organization. It is particularly illustrated here by WFT coordinators experiencing difficulties in delimiting their role and prioritising between different needs.

Further, close attention to the interaction between the change, such as the creation of a WFT coordination role, and underpinning processes and structures [38] in the municipal organisation, seems necessary. Our argument is that this study provides indications of the process of implementing the WFT coordination role faced challenges in the interaction with existing processes and structures in the municipalities. Managerial practices in the municipalities and the potential presence of a traditional hierarchical structure [26] are raised as an issue that call for further attention in relation to the implementation of a WFT coordinating role in the municipalities studied here.

### Limitations and contributions

This article is based on qualitative research and provides context dependent findings that are suitable for raising issues and questions for further research, not generalisable claims. Some of our suggestions about issues in need of further research attention in relation to WFT implementation are well known from other areas of implementation research. One example is the potential influence of organizational structures and processes on the unfolding of change implementation. However, the research conducted here directs attention to additional organisational contexts in which such conditions are argued to interact with the unfolding of change implementation. They are here empirically illustrated within the context of WFT implementation, through which more awareness and discussion on their potential impact can be brought into the debate on how to implement WFT. Similar arguments can also be applied to the influence of different elements in change designs emphasized in this article.

The collection of data for this research was conducted in municipalities being exposed to covid restrictions. However, the study participants did not address covid related issues in the interview contents used in this article.

## Conclusion

This study contributes to the field of digital health implementation by highlighting the organisational and managerial complexities associated with establishing a WFT coordinator role to support the implementation of welfare technology in municipal health- and care organisations. The study reveals that although the creation of a WFT coordinator role is widely regarded as a necessary and logical step, its actual implementation in the contexts studied here is hampered by unclear role definition, limited management support and structural barriers.

Based on the study, we will draw attention to suggestions of integrating elements from both top-down and bottom-up oriented designs to approach change in the research literature. A point being emphasised in this study is that the integration of a more top-down inspired “well-sequenced” change effort could have facilitated the implementation of the WFT coordination role. We also posit that more accountability for existing structures and work processes is necessary, being aware that it remains to be further explored how a reliance on top-down elements in change designs can be combined with the ambition to account for underpinning conditions in the organisation.

Furthermore, we will particularly emphasise that this material calls for more attention to managerial practices in interaction with WFT implementation. Our study demonstrates that an “outsourcing” of WFT implementation can take place at the managerial level. In line with the call for paying attention to underpinning structures and processes in the municipal organization, there is reason to call for more insights into how an outsourcing of WFT implementation by the managerial level can be constituted.

## Supplementary Information

Supplementary Material 1.

Supplementary Material 2.

## Data Availability

No datasets were generated or analysed during the current study.

## Abbreviations

WFT: Welfare technology
PVO: The University Hospital of Northern Norway’s Data Protection Office for Research
