# Supplementary material for: The search for successful welfare technology (WFT) implementation: Norwegian municipalities implementing a WFT coordinating role-a qualitative study
Source: BMC Health Serv Res. 2025 Jul 1;25:837. doi: 10.1186/s12913-025-13047-3 (PMC12211899; doi:10.1186/s12913-025-13047-3)
Supplement: Supplementary file 2 — Supplementary Material 2. [file 12913_2025_13047_MOESM2_ESM.pdf]

Interview guide Managers

Introduction:

The overall aim of this doctoral project is to look more closely at what happens when WFT is to be introduced in the municipal service provision and what factors come into play.

Anonymity: Names will be anonymised.

Tape recording: The interview will be tape-recorded and transcribed.

Time: The interview takes about 1 hour

**Research Questions**	**Interview Questions**	**Key words**
What are the municipalities focusing on when introducing WFT and how is the introduction process organised?	Can you tell us about your work with WFT and the processes you have been through?<br><br>Why are you introducing WFT in your municipality?<br><br>What are <u>your</u> expectations of WFT?	- Your role<br>- Strategy, Procurement, Project<br>- Anchoring<br>- User involvement<br>- Benefits<br>- Challenges to overcome
What happens in practice when WFT is introduced?	What is your experience from the implementation work?<br><br>Who came into this work first?<br><br>What have been the roles of the employees?	- Labour practices<br>- Distribution of roles<br>- Co-operation<br>- Enthusiasm in the departments and political organisation<br>- User involvement<br>- Knowledge and learning<br>- Enthusiasts<br>- Innovation/development
What are the inhibiting and facilitating factors in the introduction of WFT in municipal services?	What have you learnt along the way that you'd like to share?<br><br>What do you think about the way forward?	- Challenges faced<br>- Successes<br>- Surprises

Interview guide Project managers, IT-coordinators, System manager

Introduction:

The overall aim of this doctoral project is to look more closely at what happens when WFT is to be introduced in the municipal service provision and what factors come into play.

Anonymity: Names will be anonymised.

Tape recording: The interview will be tape-recorded and transcribed.

Time: The interview takes about 1 hour

**Research Questions**	**Interview Questions**	**Key words**
What are the municipalities focusing on when introducing WFT and how is the introduction process organised?	Can you tell us about your work with WFT and the processes you have been through?<br><br>Why are you introducing WFT in your municipality?<br><br>What are <u>your</u> expectations of WFT?	- Your role<br>- Strategy, Procurement, Project<br>- Anchoring<br>- User involvement<br>- Benefits<br>- Challenges to overcome
What happens in practice when WFT is introduced?	What is your experience from the implementation work?<br><br>Who follows up the work and how is the follow-up organised?	- Labour practice<br>- Distribution of roles<br>- Co-operation<br>- Enthusiasm in the service, professionals, users, politicians<br>- Requirements for reporting<br>- Knowledge and training
What are the inhibiting and facilitating factors in the introduction of WFT in municipal services?	What have you learnt along the way that you'd like to share?<br><br>What do you think about the way forward?	- Challenges faced<br>- Successes<br>- Surprises
